# Can Nutrition Play a Role in Ameliorating Digital Eye Strain?

**DOI:** 10.3390/nu14194005

**Published:** 2022-09-27

**Authors:** Drake W. Lem, Dennis L. Gierhart, Pinakin Gunvant Davey

**Affiliations:** 1 College of Optometry, Western University of Health Sciences, Pomona, CA 91766, USA; 2 EyePromise, LLC, Chesterfield, MO 63005, USA

**Keywords:** digital eye strain, computer vision syndrome, visual display terminal syndrome, digital asthenopia, dry eye disease, omega-3 polyunsaturated fatty acids, anthocyanins, carotenoids nutraceuticals

## Abstract

Digital eye strain is a complex, multifactorial condition that can be caused by excessive screen time exposure to various electronic devices such as smartphones, tablets, e-readers, and computers. Current literature suggests oxidative damage concomitant with a chronic pro-inflammatory state represent significant etiopathogenic mechanisms. The present review aims to discuss the potential dietary role for micronutrients with nutraceutical properties to ameliorate various ocular and vision-related symptoms associated with digital eye strain. For ocular surface dysfunction, enhanced anti-inflammatory benefits with omega-3 polyunsaturated fatty acids have been well documented for treatment of dry eye disease. The anti-oxidative and immunosuppressive properties of anthocyanin phytochemicals may also confer protective effects against visually induced cognitive stress and digital asthenopia. Meanwhile, nutraceutical strategies involving xanthophyll macular carotenoids demonstrate enhanced cognitive functioning and overall visual performance that aids digital eye strain. Collectively, preliminary findings seem to offer a strong line of evidence to substantiate the need for additional randomized controlled trials aimed at treating digital eye strain with adjunctive nutraceutical strategies. Further RCT and comparisons on commercially available nutritional supplements are needed to quantify the clinical benefits.

## 1. Introduction

Digital Eye Strain is a multifactorial disease which encompass a large group of ocular and vision-related symptoms that can be attributed to prolonged and extended use of smartphones, tablets, e-readers, and computers [1,2]. In recent years, these electronic devices have become nearly ubiquitous in modern society and have given way to an ever-increasing global dependence upon their application across personal, educational, and occupational settings. While computers and smartphones may serve to enhance our daily lives and activities, the American Optometric Association found that as few as two hours of uninterrupted screen time exposure is sufficient for the onset of both ocular discomfort and vision-related problems to develop [2]. Long-term implications associated with digital eye strain have yet to be elucidated, however, a large body of evidence has already demonstrated an array of harmful physiological effects associated with greater time spent using digital display devices [3,4,5,6,7]. In lieu of this, one can safely predict the current global COVID-19 pandemic will further exacerbate the prevalence of digital eye strain into epidemic proportions affecting nearly all age groups.

In this digital era of increasing screen time habits, or time spent looking at these devices and omnipresent exposure, the incidence of screen-induced ocular health issues and visual discomfort will continue to present major public health issues [4,5,6]. Some reports estimate the overall prevalence may impact up to 90% of individuals in some populations making this an endemic problem that will require our utmost attention [6,8,9,10,11]. Recently, the Vision Council found that >80% of adults in the United States far exceeded the two hour minimum of daily use associated with greater risk for the onset of digital eye strain symptoms [2,12]. In fact, working-age adults are estimated to spend an average of seven hours daily using computers just for their profession [2], of which, roughly 60% reported experiencing symptoms of screen-induced visual stress [12]. As one would expect, similar habits are seen among children and adolescents wherein >70% regularly exceed two hours of daily exposure [12,13,14] and are more often using two or more devices simultaneously [15,16,17,18,19]. In consequence, multi-tasking with more than one electronic device often leads to further risk of developing symptoms and greater incidence of visual fatigue [6,12,20]. Moreover, several reports also show that both adults and adolescents routinely use smartphones and hand-held devices roughly one hour before going to sleep and immediately upon awakening in the morning [15,16,18,21,22].

While it is important to note, the physiological implications are not uniform across all electronic devices with digital display technology; for example, it appears there are distinguishable patterns between symptom profiles associated with the overuse of computers versus smartphones [3,16,22,23]. Hence, this may explain, at least in part, the high prevalence of digital eye strain amongst several types of individuals, including computer users [24,25], visual display terminal or “teleworkers” [3,26,27,28], technicians [29], university students and young adults [15,30,31,32,33,34,35,36], as well as children and adolescents [17,19,25,37,38,39,40,41,42,43]. Nonetheless, there is considerable evidence that substantiate the positive relationship between total amount of time spent using digital devices and overall risk of developing symptoms associated with digital eye strain [6,7,20,31,44,45].

### 1.1. Ocular and Vision-Related Symptoms

Traditionally, the effects of digital eye strain have been referred to interchangeably as computer vision syndrome (CVS) [1,2,32,46,47], as well as digital asthenopia [29,48], and vision display terminal (VDT) syndrome [3,24]. Commonly reported symptoms include eyestrain, eye soreness, headaches, blurred vision, diplopia, and dry eyes [4,5,6,8,46,49,50]. These ocular effects can be categorized according to: (1) external symptoms commonly associated with dry eye disease regarding changes in ocular surface homeostasis [2,15,49,51,52,53,54]; and (2) internal effects relating to aesthenopic symptoms and visual function impairment [6,8,24,55,56]. Although helpful, these distinctions are not mutually exclusive measures of disease etiology due to the subjective nature of visual sensory processing and high degree of variability among patient-reported symptoms. In consequence, the diagnostic parameters used to characterize digital eye strain often vary among available reports.

While the pathophysiology extends beyond the scope of the present review, it is important to briefly discuss the various contributing factors as they relate to the ability of nutrition in ameliorating symptoms associated with digital eye strain [6,44,49,57]. A number of excellent reviews which have discussed several putative mechanisms in more detail along with the spectrum of physiological effects can be found elsewhere [4,5,6,8,46,54,58,59].

#### 1.1.1. Dry Eye Disease

Dry eye is among the most common ocular complaint reported by individuals with digital eye strain [9,51,52,60,61,62]. Symptoms often range from irritation, burning, and stinging, as well as epiphora and foreign body sensations [63,64]. It has been well-documented that greater screen time behaviors represent a major component in developing symptoms of dry eye, often associated with lacrimal gland dysfunction and signs of evaporative dry eye disease [3,27,44,58,60,61,62,65,66,67,68]. In particular, computer usage significantly influence various dynamics of blinking patterns (such as frequency, amplitude, and complete vs. incomplete) thereby further increasing the rate of evaporation and exacerbating tear film instability [18,23,54,58,68,69,70,71,72,73,74,75]. The combination of sub-optimal tear production and excessive evaporation can lead to tear hyperosmolarity with subsequent inflammation of the epithelial surface [76,77,78,79,80].

Dry eye-related symptoms of digital eye strain may also be attributed to meibomian gland dysfunction (MGD) [81,82,83,84,85,86,87,88,89]. Normal sebum production from these glands serves as an important role in preserving ocular surface homeostasis by regulating evaporation of the tear film. Furthermore normal secretion and movement of meibomian glands depends on adequate blink dynamics, in fact, some reports indicate that dysfunction of meibomian glands may be responsible for triggering initial inflammatory response mechanisms in consequence of abnormal sebum production [82,84,85]. Furthermore, greater time spent using electronic devices have been shown to positively correlate with diagnostic parameters for MGD including meibum quality score, lipid margin abnormalities, and meibography gland drop out [88,89,90,91].

Moreover, it appears that a cascade of pro-inflammatory mechanisms which perturb homeostasis of the ocular surface are implicated in the onset of dry eye symptoms and ocular discomfort associated with digital eye strain. Among patients with dry eye disease, conjunctival and tear fluid samples provide indications of a pro-inflammatory condition marked by increased concentrations of late lipid peroxidation markers concomitant with reductions in endogenous antioxidant enzymes [92,93]. Early pathophysiology likely involves a vicious cycle between pro-oxidative and pro-inflammatory mechanisms which further contribute to worsening dysfunction of the ocular surface [49,59,66,67,81,93,94,95,96,97,98,99,100].

One school of thought suggests prolonged exposure to digital displays may serve an important role in exacerbating the extent of oxidative damage to various structures of the eye [59,88,89,101]. Peak spectral emission (visible blue light, 400–490 nm) from light-emitting diodes commonly used in digital display technology have been implicated with causing photo-oxidative damage to the outer retina, that is photoreceptors and retinal pigment epithelial cells [102,103]. It is known that short-wavelength (blue) light is of high energy and capable of proliferating reactive oxygen species (ROS) formation in a time-dependent manner [101,102]. Additionally, oxidative damage and apoptosis brought on by blue light irradiation within ocular surface tissues have been implicated with clinical manifestations of dry eye disease [59,88,89,104].

#### 1.1.2. Asthenopia

With increasing screen time behavior, digital asthenopia (i.e., eye strain or fatigue) remains the most common visual complaint alongside blurred and double vision paired with headaches and ocular soreness [11,24,42,48,55,105,106,107,108]. Difficulty focusing between working distances can be attributed to accommodative and vergence-related stress in consequence of uncorrected refractive error or continuous fixation at close-viewing distances [6,8,24,55,56]. In comparison to reading printed text, using hand-held devices such as smartphones and tablets, impose a greater burden on ocular muscles leading to greater recession in near point of convergence and reductions in accommodative function [109,110,111,112]. In many cases, aesthenopic symptoms seem to emerge over time when the cognitive demands for a visual task overwhelm the individual’s ability to perform them comfortably [1,2,31,34,113,114,115,116]. For instance, the visual demands of uninterrupted computer work can manifest as headaches and ocular discomfort due to glare and increased squinting.

### 1.2. Extraocular Symptoms

Often presenting as secondary perturbations that may arise in conjunction with vision-related symptoms, clinical manifestations of digital eye strain are not exclusive to our visual system tissue. For instance, office workers commonly report experiencing myofascial pain and discomfort in the neck, shoulders, and upper back regions [3,5,46,117]. Indications of musculoskeletal symptoms appear strongly associated with the postural demands of computer work, in addition to poor ergonomic practices and extended periods of physical inactivity [3,6,46,49,50,118,119].

On the other hand, greater use of hand-held electronic devices have also been associated with the preponderance of psychological disorders [43,120,121,122,123,124,125,126] and disruption in circadian rhythms [21,22,46,117,127,128,129]. It is well-documented that excessive screen time behaviors before bedtime may significantly alter the sleep-wake cycle which can lead to significant disturbances in sleeping patterns. Particularly among adolescents and younger adults, reports of digital eye strain are often associated with sleeping disorders such as insomnia and excessive daytime sleepiness [21,22,121,122,127,130,131]. Consequentially, chronic patterns of sleep loss and circadian misalignment ascribed with an evening chronotype are also linked to greater psychosocial stress paired with increased systemic markers of stress-related hormones [132,133,134,135]. Regular behaviors of excessive screen time activities among students are also strongly associated with greater risk of developing signs of anxiety and depression [120,123,124,126]. One school of thought suggests dry-eye related symptoms may help to explain, at least in part, some similarities observed between sleeping disorders and changes in mental health condition associated with overuse of hand-held devices in younger populations [68,123,136,137,138,139].

Moreover, chronic exposure to psychological stressors have been linked with triggering a pro-oxidative state throughout the body, and it appears that ameliorating systemic oxidative stress may considerably reduce measures of psychological stress as well [140]. Given the relationship between proper dietary behaviors and overall well-being, it is no surprise that regular consumption of nutraceuticals and foods rich in antioxidants (i.e., fresh fruits, leafy green vegetables, and fish) may offer protection against elements of biopsychosocial deterioration [141,142,143,144,145].

Nutraceuticals are dietary supplements that have greater amounts of nutrients that are naturally present in nature and consumed by individuals as routine part of diet. The nutraceuticals are pharmaceutical-grade supplements that have the potential of modulating disease pathways or disease state. Thus, further reinforcing the potential therapeutic application for nutrition to ameliorate the purported systemic oxidative condition associated with digital eye strain. However, they can only be marketed to support the structure or function of the body and the label of the nutraceuticals or dietary supplements includes disclosure that they are not intended to diagnose, treat, cure, or prevent diseases and they are not evaluated by the Food and Drug Administration (FDA) in the US.

## 2. Omega-3 Fatty Acids

Given that a core etiopathogenic mechanism of dry eye-related symptoms involve a chronic pro-inflammatory state, research has been focused on investigating adjunctive nutraceutical strategies aimed at targeting this component of ocular surface dysfunction. Due to their inherent anti-inflammatory properties and immunomodulatory potential, considerable research has been focused on the role of omega-3 polyunsaturated fatty acids (PUFAs) [146,147,148,149,150,151,152]. By increasing dietary consumption of omega-3 fatty acids compared to omega-6 fatty acids, clinical reports have demonstrated some ability to regulate the body’s inflammatory state by attenuating pro-inflammatory mediators [146,148]. Omega-3 PUFAs also serve an important role in the prevention of chronic systemic conditions such as cardiovascular disease [152,153,154,155], in addition to exerting protective ocular effects against cataracts [156,157,158] and age-related macular degeneration (AMD) [159,160,161,162].

For the management of ocular surface symptoms in digital eye strain, the capacity for omega-3 fatty acids to offer clinical benefits against the underlying mechanisms of dry eye disease is supported by robust scientific evidence [76,146,147,148,150,152,163,164,165,166,167,168,169]. In randomized clinical trials, short-term dietary supplementation with omega-3 PUFAs demonstrated enhanced therapeutic benefits in patients with mild-to-moderate dry eye disease (Table 1) [170,171,172,173,174,175,176,177]. A systematic review and meta-analysis found that patients receiving omega-3 PUFAs saw significantly better improvements in tear evaporation, tear osmolarity, and severity of dry eye symptoms compared with placebo [151]. Odds ratio (OR) for improvements in tear break-up time (TBUT) were significantly greater among those in the active treatment groups (OR: 8.72; 95% CI: 4.73–16.09; *p* < 0.001) [151]. Multivariate analyses performed by separate meta-analyses seem to mirror these findings, wherein short-term supplementation was also associated with increased tear production and secretion from lacrimal glands (Schirmer’s test scores; *p* < 0.001) [167,168]. Based on the available evidence from clinical trials in patients with dry eye disease, one can postulate that nutraceutical strategies involving omega-3 fatty acids would likely alleviate similar signs of ocular dysfunction brought on by prolonged digital device use. This would particularly be important for individuals that have sub-optimal tear film dynamics and predisposed to dry eye disease and involved in significant activities with digital devices.

To date, short-term omega-3 fatty acid supplementation has demonstrated promising results to offer a similar degree of clinical benefit for symptomatic patients with digital eye strain (Table 1) [171]. Prospective studies involving younger adults with computer vision syndrome (≥3 h/day computer use) demonstrated significant improvements in objective measures of inherent tear film stability and subjective dry eye symptoms in as few as 45 days of supplementation [171,178]. Marked improvements in Nelson grading scores upon impression cytology seem to mirror these findings, providing further evidence of the nutraceutical benefits of omega-3 fatty acids to promote healing of the conjunctival epithelium [169,171,178]. Given that hyperosmotic stress plays an important role in causing damage to the ocular surface, these observations wherein short-term supplementation produced a profound normalization in tear tonicity represent clinically meaningful effects to improve the severity of dry eye [179,180]. Similarly, these improvements in tear film stability may also be attributed to the nutraceutical effect of omega-3 fatty acids on ameliorating signs of MGD [81,82,83,84,85,87]. Given the importance of sebum production in maintaining proper stability of the tear film layer, the potential for these micronutrients to improve meibum composition scores and meibomian gland secretions should not be overlooked [174,175,177]. It should be noted that greater aqueous tear production was also observed following three months of supplementation in patients with computer vision syndrome [171], consistent with reports in dry eye disease [151,167,168].

For treatment of digital eye strain, the growing number of preliminary reports offer promising clinical evidence substantiating the capacity for omega-3 fatty acids to ameliorate signs of ocular surface dysfunction and dry eye-related symptoms. However, it is important to acknowledge a recent systematic review and meta-analysis by Singh et al. [181] that concluded that there is low-certainty evidence of benefits of omega-3 supplementation on reduction of dry eye symptoms in individuals that are symptomatic computer users.

## 3. Phytochemicals

Flavonoids, a large group of polyphenolic compounds found in a variety of plant species, demonstrate unique medicinal properties for ocular health to support the rationale for their inclusion in nutritional strategies for ameliorating signs of digital eye strain [182]. Among them, anthocyanins have been widely used in traditional medicine specifically for improving scotopic vision and alleviating eye fatigue in older adults [183,184,185]. These phytochemicals demonstrate remarkable anti-oxidative, anti-inflammatory, and immunomodulating properties [182,183,184,185,186,187,188,189].

Derived from fruits and leafy vegetables, the protective benefit afforded by these nutraceuticals often varies based on the composition of anthocyanin complexes acquired from the dietary source [183,184]. For instance, vasorelaxant properties of anthocyanin-rich extracts derived from blackcurrant (*Ribes nigrum* L.) have been shown to improve retinal microcirculation in normal tension glaucoma [190,191]. On the other hand, bilberry (*Vaccinium myrtillus* L.) extracts containing cyanidin-3-glycoside promote rhodopsin regeneration in the retina during visual phototransduction cascade [192]. Several major anthocyanins also exhibit enhanced antioxidant capacity in ocular tissue by attenuating light-induced oxidative stress and lipid peroxidation [183,184,192]. In lieu of the absence of recommended daily intake values established by the US Food & Drug Administration, oral supplementation may be the best dietary strategy to ensure sufficient acquisition of these micronutrients to promote optimal visual performance.

Prospective interventional studies containing anthocyanin extracts in formulation seem to demonstrate therapeutic protection against several asthenopic symptoms in patients with heavy screen time behaviors (Table 2) [193,194,195,196,197,198,199,200,201,202]. While the relationship between desktop computers and digital eye strain symptoms is clear, the putative implications of mobile smartphones and handheld devices on developing similar ocular discomforts and binocular vision stress are still under investigation [3,5,16,22,23,56]. Studies have shown less than one hour of smartphone or tablet use is sufficient to induce eye fatigue and non-strabismic accommodative alterations in younger adults [25,51,112,203,204,205]. It may not appear significant at first glance, however a 1.00 diopter reduction in accommodative amplitude following such a brief period of exposure raises particular concern regarding more prolonged durations of use [56,112,205]. Although the exact mechanism by which these devices may disrupt accommodation and vergence systems is unclear, some suggest increased cognitive demands from multitasking coupled with varying font size and contrast may be responsible for these visual anomalies.

Dietary supplementation with anthocyanins, either alone or in combination with other nutraceuticals, offered some degree of benefit in accommodative function following a brief VDT task emulating the visual load induced by handheld devices and near-vision work (Table 2) [193,194,197,199,200,206]. Among those receiving only standard bilberry extract, researchers saw significantly improved values in the high-frequency component of accommodative microfluctuation, indicating greater refractive power and ciliary muscle activity after briefly playing iPhone game [193,207,208,209]. Perhaps by improving microcirculatory dynamics within the relevant ocular muscle groups, dietary intake of the test food is suggested to relieve tonic accommodation induced by mobile devices [193,210]. Additional reports of ciliary smooth muscle relaxation seem to further corroborate these findings, wherein significant improvement in pupillary response was also observed following short-term nutraceutical intervention [197,199]. Given that mydriasis and reduced pupillary constriction can occur after only 20 min of handheld device use, these findings offer clinically meaningful evidence whereby anthocyanins may inhibit transient refractive alterations in accommodative asthenopia [199,209,211,212].

Prospective studies are consistent wherein anthocyanin-rich extracts may also offer therapeutic mitigation for individuals experiencing subjective symptoms of visual discomfort and presbyopia related to digital eye strain. Current reports indicate intake of anthocyanins led marked improvements in sensations of ‘tired eyes’, ‘eye fatigue’, and blurred vision caused by watching a movie on iPad or playing handheld video games for up to one hour [194,195,198,200]. These findings are encouraging given significant reductions in binocular accommodative amplitude have also been observed with equivalent periods of short-term smartphone and computer use [17,75,112,213,214,215]. Appositely, some suggest the accommodative insufficiency may be largely responsible for the pervasive number of visual disturbances and ocular fatigue symptoms [216,217,218,219,220]. However, the effects of anthocyanin supplementation on near point of accommodation are inconclusive among current reports [194,200]; suggesting the inclusion of additional micronutrients may explain the positive effects reported on accommodative amplitude [200].

On the other hand, patients receiving only anthocyanins from bilberry extract show improved subjective symptom scores concomitant with critical flicker-fusion frequency (CFF) [194]. An established indicator of visual performance regarding temporal resolution, attenuation of CFF response has been ascribed to mental fatigue and decreased retinogeniculate activity (i.e., asthenopia) [109,110,221,222,223]. Consequential decline in this parameter while using computers and handheld devices is believed to correlate with worsening eye fatigue and complaints of visual discomfort [211,224]. Surprisingly, a recent systematic review and meta-analysis, did not report a statistically significant effect on visual fatigue using pooled data from several additional anthocyanin-containing berry extract clinical trials [181]. Researchers suggest the discrepancy may be attributed, at least in part, to differences in study design regarding visual fatigue questionnaires. Despite this, these findings may provide early justification for the potential role of anthocyanins to alleviate symptoms of cognitive fatigue associated with digital eye strain.

Furthermore, preliminary studies also suggest dietary intervention with these phytochemicals may be beneficial for signs of ocular surface dysfunction underlying dry eye-related symptoms as well [196,198,206]. Short-term consumption of anthocyanin-rich extracts from either bilberry or maqui berry (*Aristotelia chilensis*) produced a significant increase in tear secretion volume after four weeks [196,198]. Researchers suggest the antioxidative properties of anthocyanin complexes may be responsible for these improvements in lacrimal fluid secretion. In fact, a major anthocyanin found in both bilberry and maqui berry extracts, delphinidin-3,5-O-diglucoside is known to inhibit free radical formation thereby attenuating tissue dysfunction in the lacrimal glands and corneal epithelium [184,225,226]. Congruously, one study also saw enhancement in potential antioxidant capacity (BAP/d-ROMs ratio) following dietary intervention with standardized bilberry extract [196]. These findings seem to indicate anthocyanins may offer additional, synergistic protection against mechanisms of oxidative stress and changes in cellular redox homeostasis believed to be associated with digital eye strain.

Resveratrol (3,5,4′-trihydroxy-*trans*-stilbene), often found in grape skin, is known to be a potent antioxidant with anti-inflammatory properties that may benefit numerous ocular diseases like glaucoma, cataract, diabetic retinopathy, and AMD [227,228]. In vitro and in vivo animal model studies have looked at the biological effects of resveratrol and proposed several mechanisms of action [227]. However, there is a paucity of clinical evidence for resveratrol supplementation in treating digital eye strain and future trials should evaluate this further and my show benefits from including this phytochemical in formulation [227].

## 4. Carotenoids

In consequence of its extraordinarily high metabolic demands and inherent exposure to visible light spectrum, the retinal tissue is known to be particularly vulnerable to free radical formation and subsequent activation of pro-inflammatory mechanisms [229,230,231]. Hence, a major concern is the potential for long-term phototoxicity culminating from pernicious blue light (400–490 nm) emitted from LED-backlight modules utilized in most consumer electronics [232,233,234]. In a time-dependent manner, highly reactive short wavelength (blue) light is capable of proliferating formation of reactive oxygen species (ROS) in the most sensitive layers of the neurosensory retina [101,102,231,235,236,237,238,239,240,241]. Fortunately, the human eye possess an intrinsic optical filter comprised of dietary carotenoid pigments, which demonstrate enhanced neuroprotection against photo-oxidative injury brought on by aberrant blue light exposure [231,236,237,238,239,242,243,244,245,246,247,248,249].

Xanthophyll carotenoids lutein and zeaxanthin, as well as *meso*-zeaxanthin an isomeric conversion of lutein known as macular carotenoids, serve a fundamental role in maintaining retinal integrity in addition to promoting optimal central visual acuity [231,236,250]. Given their unique distribution in the central fovea, together they comprise the macular pigment which is believed to preserve local tissue through two primary mechanisms: (1) by absorbing harmful blue light; and (2) actively neutralizing free radicals thereby ameliorating further oxidative damage [236,237,238,239,242,243,244]. By attenuating exposure to high-energy wavelengths of light, the macular pigment’s peak wavelength of absorption (~460 nm) serves to limit further ROS generated by photosensitizers (i.e., rods and cones) in outer retina [237,240,250]. In addition to their potent anti-oxidative properties, macular carotenoids may also enhance total antioxidant capacity by promoting endogenous antioxidant defense mechanisms [231,239,246,247,248,249].

Since humans have lost the ability to naturally synthesize lutein and zeaxanthin in the body [236,251,252], the primary method for acquiring these protective micronutrients is by consuming carotenoid-rich foods like spinach, kale, and other cruciferous leafy green vegetables, along with corn, carrots, orange bell peppers and egg yolk [231,236,243,249,253,254]. Diminution of macular xanthophylls, evidenced by macular pigment optical density (MPOD) depletion, serves as a clinically relevant biomarker linked to increased risk of incident retinopathy and visual function impairment [230,231,244,246,247,248,249,255]. Meanwhile, an abundance of clinical trials have indicated remarkable therapeutic benefits by supplementing their levels via adjunctive carotenoid vitamin therapy in diabetic retinopathy, open-angle glaucoma, and most notably, age-related macular degeneration (AMD) [231,236,242,247,248,249,250,251,256,257,258,259,260,261,262,263,264,265,266].

While the scientific rationale for dietary strategies involving these nutraceuticals is quite clear, there is limited evidence pertaining to xanthophyll supplementation in treating digital eye strain available to date [200,201,206,267,268]. Traditionally, carotenoids by themselves did not appear to alleviate dry eye or signs of ocular fatigue so they were often combined with anthocyanins and other antioxidants in multivitamin formula to improve these symptoms [199,200,201,206,267]. However, alterations in macular pigment status have been posited as a surrogate for visual performance in both healthy and diseased states [231,269]. Maintaining greater MPOD levels have been shown to improve several functional outcomes that likely correspond with symptoms of asthenopia, including: light sensitivity (photophobia) [270,271], glare disability [269,272,273], and photostress recovery [269,272,273,274], along with visual temporal resolution [275,276,277] and contrast sensitivity [269,278,279,280,281]. Baseline correlations from available reports indicate MPOD was significantly associated with eye strain frequency, as well as psychological stress scores, in addition to these visual outcome measures [268,282,283]. Therefore, evidence from preliminary trials wherein carotenoid vitamin therapy is found to enhance macular pigment concentrations with concomitant benefits in visual performance, may be clinically relevant for treating individuals with digital eye strain (Table 3).

A major component in digital asthenopia are the effects of glare, which have been shown to significantly influence reading speed and remain among the most pervasive screen-related symptoms of digital eye strain [8,21,57,128,284,285,286,287,288,289]. Visual consequences engendered by the glare source can originate directly from LED-displays or environmental lighting conditions [6,8,46,50,57]. In clinical trials lasting up to 12 months, oral supplementation containing all three macular carotenoids offered remarkable improvement on composite measures of visual performance in glare conditions [268,282]. While similar therapeutic benefits in disability glare thresholds and photostress recovery have been shown in earlier reports [260,272,273,274,290,291,292], researchers suggest the level of improvement in both glare measures were strongly associated with increased MPOD concentrations in a dose–response relationship [268,274,282]. A plausible mechanism by which MPOD levels advantageously influence these aspects of glare sensitivity are likely due to selective filtration. Indeed, the functional capacity of the macular pigments to preferentially absorb short wavelength (blue) light abate the influence of chromatic aberration thereby modifying the image formed at the level of perception [268,272,273,274,278,282,293,294]. Moreover, by filtering scattered light at the pre-receptoral level, enhancement of MPOD likely attenuates the proportion of bleached photopigment exposed to bright light conditions leading to subsequent improvements in recovery speed and visual capacity [268,272,282].

As visual fatigue often ensues after prolonged durations of digital device use, reports suggest long-term carotenoid vitamin therapy may also elicit synergic neuroprotection whereby increasing their concentrations in the local tissue seemed to enhance mechanisms of physiological processing in the visual system [268,282]. This may explain, at least in part, corresponding changes in contrast sensitivity function which provide a comprehensive assessment of spatial sensitivity [268,295,296]. One school of thought strongly suggest greater MPOD levels likely represent an essential condition that must be met for commensurate change in visual performance to become clinically apparent; for example, measurable improvements in contrast sensitivity may only become significant once the macular pigment had been maintained at higher concentrations for some period of time [246,249,260,293,297].

The neurophysiological basis for contrast sensitivity may further substantiate these visual benefits afforded by carotenoid vitamin therapy given their exceptional antioxidant proficiency in tissues under extreme metabolic stress [231,239,246,280]. Perhaps by ameliorating mitochondrial dysfunction in the neurosensory retina, dietary augmentation of macular xanthophylls facilitate an improved redox state thereby enhancing metabolic efficiency of the visual cycle [268,280,295]. Interestingly, it appears that MPOD status is significantly associated with lateral inhibition sensitivity, the core mechanism underlying contrast sensitivity thresholds which rely upon the visual system’s propensity for edge detection [280,298,299]. It may be the nutraceutical potential of macular carotenoids to augment homeostatic redox control pathways consequently optimize nitric oxide levels in local synaptic networks [277,300,301]. A redox-sensitive neurotransmitter, nitric oxide has been implicated with improving lateral inhibitory processes by enhancing signal-to-noise ratio; ultimately resulting in greater contrast sensitivity [277,300,301].

The “neural efficiency” hypothesis has also been posited as a plausible explanation for these findings whereby measures of temporal visual function appear strongly associated with MPOD status [268,276]. It is well accepted that temporal metrics are reliable indications of visual processing capacity [109,110,221,222,223]. Reports indicate the metabolic effects of xanthophyll carotenoids on neural encoding processes may extend beyond the retina and influence visual processing at various levels along the retinogeniculate pathway [247,248,249,302,303,304]. In fact, lutein and zeaxanthin appear to preferentially accumulate in the brain, specifically within regions under extremely high metabolic activity and subsequent oxygen tension [304,305,306,307,308,309]. Lutein and zeaxanthin deposits in the brain have also been found to correlate significantly with MPOD levels [309]. Following this line of reasoning, it is likely the cumulative increase in both exogenous and endogenous antioxidant capacity with long-term carotenoid vitamin therapy would yield subsequent neuroprotective benefits at the post-receptoral level [246,247,248,249,268,280,295,304,310]. These findings seem to corroborate this hypothesis whereby those with higher MPOD appear to process visual stimuli more effectively in their retina; particularly in glare conditions [268,282]. Following a repeated-exposure measure to emulate the dynamics of photostress recovery, substantially faster and more consistent visual recovery performance was observed among those with greater MPOD [282]. Hence, the therapeutic potential for carotenoid vitamin therapy in digital eye strain to augment macular pigment concentrations appear to facilitate an optimal state of visual adaptation under exceedingly bright light conditions (i.e., LED displays) [268,278,282,283,293,295,311].

Furthermore, therapeutic strategies aimed at enhancing macular xanthophyll concentrations are thought to play an important role in alleviating psychological stress as well as promoting both physical and mental well-being. Following long-term supplementation with all three macular carotenoids, clinical studies observed remarkable benefits in serum cortisol, reduced anxiety scores, and improvement in overall sleep quality among healthy young adults [268,283]. Researchers suggest the observed effect on cortisol reduction following carotenoid vitamin therapy may involve anti-inflammatory actions within local neurosensory tissues thereby counteracting the physiological implications associated with the stress response [283,312]. For example, previous reports have reported marked inhibition of the endogenous antioxidant system in consequence of stress-induced corticosteroid production [312,313]. Thus, the neuroprotective capacity of these macular carotenoids to reduce local oxidation and inflammation may explain, at least in part, these systemic effects observed on serum cortisol levels and psychological stress.

Given the implications of blue light exposure on circadian rhythm disturbances, the potential for carotenoid vitamin therapy to elicit meaningful improvements on sleep outcomes likely represent clinically relevant findings that warrant further investigation [268]. Early reports involving university students with excessive screen time exposure (≥6 h/day) reported significant improvements in overall sleep quality scores (Pittsburgh Sleep Quality index) following six-months of nutraceutical intervention with macular carotenoids [131,268]. Among the most prevalent age groups with heavy screen time behaviors before bed, adolescents and young adults represent growing populations that may be particularly vulnerable to psychosocial implications (sleeping disorders, emotional distress, interpersonal anxiety) associated with digital eye strain [128,129,314,315]. Additionally, while many smartphones market the ability to limit short-wavelength light exposure at night, high-quality clinical evidence to substantiate blue-light attenuating filters along with equivalent ophthalmic lenses to improve sleep quality among healthy individuals is limited and controversial at best [26,128,129,181,316,317,318,319].

Lastly, while clinical trials have shown that oral supplementation containing all three macular xanthophylls offer protection against mechanisms of retinal neurodegeneration brought on by blue light irradiation, it is unclear whether these nutraceuticals may prevent accommodative asthenopia in digital eye strain. However, carotenoid vitamin therapy containing a similar xanthophyll carotenoid known as astaxanthin has demonstrated ocular benefits on retinal microcirculatory hemodynamics [199,320,321,322,323,324,325]. Astaxanthin also possess the highest degree of antioxidant capacity among carotenoid molecules [326]. Importantly, one study observed significant improvements in accommodative amplitude among VDT workers following 4 weeks supplementation containing only astaxanthin [325]. Improved ciliary body function and reduced eye fatigue are believed to result from astaxanthin’s ability to augment blood flow to the ciliary muscles [199,325].

**Table 3:** Characteristics of the randomized clinical trials using carotenoids.

Author (Year)	Participants	Duration	No. of Groups	Interventions per Day	Results
Kan (2020) [206]	360 adults with DES; aged (38.3 ± 8.3) years in China	90 days	4	12 mg L + 1.2 mg Z; 20 mg L + 2 mg Z; 28 mg L + 2.8 mg Z; placebo	Significant improvement in TBUT, Schirmer’s test, and eye fatigue symptoms (*p* < 0.01, for all)
Kawabata (2011) [201]	20 adults with heavy VDT use; aged (25.2 ± 1.2) years in Japan	4 weeks	2	17.5 mg L (*multivitamin*); placebo	Safely improved subjective complaints of asthenopia and mental fatigue from VDTs
Kizawa (2021) [199]	44 adults with DES; aged (36.6 ± 9.1) years in Japan	6 weeks	2	5 mg L + 3 mg Ax (*multivitamin*); placebo	Ameliorated reduction in accommodative function and visual performance (both *p* < 0.05)
Kono (2014) [200]	48 adults with eye strain; aged (52.8 ± 0.9) years in Japan	4 weeks	2	10 mg L + 4 mg Ax (*multivitamin*); placebo	Protection against accommodative amplitude decline from VDT use (*p* < 0.05)
Ma (2009) [295]	37 adults with DES; aged (24.8 ± 2.0) years in China	12 weeks	3	6 mg L; 12 mg L; placebo	Higher intake of lutein may offer enhanced benefit in visual performance measures
Nagaki (2002) [325]	26 adults with VDT use; aged (47.7 ± 4.4) years in Japan	4 weeks	2	5 mg Ax; placebo	Marked increase in accommodative amplitude (*p* < 0.01)
Stringham (2016) [282]	59 healthy young adults; aged (21.7 ± 1.0) years in USA	12 months	3	10 mg L + 1 mg Z + 1 mg MZ; 20 mg L + 2 mg Z + 2 mg MZ; placebo	Significant increase in MPOD resulted in improved PSR and DG (*p* < 0.001, for all)
Stringham (2017) [268]	48 healthy adults with +6 h/day screen time; aged (21.2) years in USA	6 months	2	20 mg L + 2.5 mg Z + 1.5 mg MZ; placebo	MPOD increased significantly along with enhanced visual performance measures and sleep quality (*p* < 0.05, for all)
Stringham (2018) [283]	59 healthy young adults; aged (21.5) years in USA	12 months	3	10.86 mg L + 2.27 mg Z-MZ isomers; 22.3 mg L + 4.7 Z-MZ isomers; placebo	Statistically significant relationship between increased MPOD and reductions in serum cortisol (*p* < 0.001) and psychological stress (*p* = 0.002)

Abbreviations: DES, digital eye strain; L, lutein; Z, zeaxanthin; TBUT, tear break-up time; VDT, visual display terminal; Ax, astaxanthin; MZ, *meso*-zeaxanthin; MPOD, macular pigment optical density; PSR, photostress recovery; DG, disability glare.

In recent years, there is a push towards Precision Medicine with National Institute of Health spear heading this initiative [327]. Precision medicine, also known as Individualized Medicine, although has its roots in oncology and treatment of various cancers, the protocols established allows for applications into chronic diseases [328]. In a recent study Kan et al., [329] have addressed the use of lutein-based formula in the amelioration of symptoms caused by eye fatigue and dry eye. Using artificial intelligence strategies particularly extreme gradient boosting (XGBoost) algorithm. They identified 504 features that included patient demographics, eye related indexes, blood biomarkers and dietary habits [329]. The features that were found to be most predictive of the Visual Health Score that represented the overall eye fatigue included measurement of Macular Pigment Optical Density, Schirmer’s test, visuognosis persistence, eye fatigue symptoms. Using these features and the XGBoost algorithm they could predict the dose needed to relieve symptoms of asthenopia. Further they evaluated their XGboost personalized medicine algorithm they could predict at baseline individuals that needed a 14 mg or could take a lower dose [329]. Strategies like this [329] and objective in vivo measurement of individual carotenoids [330,331] will aid in taking us away from current conventional one size fits all strategy to contemporary practice.

In a recent systematic review and meta-analysis Singh et al., [181] evaluated various interventions for management of computer vision syndrome. They found low certainty evidence that suggested the use of omega-3 supplementation in reducing dry eye symptoms, and low certainty evidence of carotenoids improving CFF compared to a placebo [181]. Singh et al., did not report a statistically significant effect on visual fatigue using pooled data from several additional anthocyanin-containing berry extract clinical trials [181]. They report that there were 12 studies that had published outcomes whereas 24 studies were still ongoing [181]. It is important to acknowledge that there is shortage of large scale RCTs that have evaluated the benefits of nutritional supplements on digital eye strain and the current analysis may in part be erroneous due to premature meta-analysis of published data. Albeit we agree that greater level of evidence is indeed needed.

Equally important is to acknowledge is the complexity of the digital eye strain and the syndromic nature of the multifactorial condition. This is not a syndrome just related to optical phenomenon’s but includes oculo-physiological changes and systemic physiological changes are observed. It is further complicated by the absence of objective biomarkers of fatigue, asthenopia and digital eye strain and the use surveys and questionnaires are a poor surrogate at best and may not be able to capture the issues and extent of improvement or changes by various interventions. Currently, clinicians often recommend for office workers and heavy computer users to shift their field of vision, every 20 min, toward an object 20 m away for roughly 20 s [1,2,8,12]. While adhering to this “20-20-20” rule may certainly be beneficial, reports suggest that improved ergonomic practices alone may not be sufficient to properly alleviate the array of ocular and visual symptoms brought on by these digital devices, in addition to hand-held smartphones and tablets.

There is significant molecular and mechanistic basis that purports the use of nutritional intervention, particularly the use of omega-3, anthocyanins, and carotenoids in management of digital eye strain. Perhaps well-suited for managing dry eye symptoms, short-term supplementation with omega-3 fatty acids demonstrate enhanced capacity to ameliorate pro-inflammatory mechanisms of ocular surface dysfunction and MGD [151,167,168,169,170,171,172,173,174,175,176,177,178]. A growing body of evidence indicates a potential role for anthocyanins to provide dietary benefits against aesthenopic symptoms along with visual fatigue brought on by hand-held devices and prolonged near-vision work [193,194,195,196,197,198,199,200,202,206]. Adjunctive carotenoid vitamin therapy offers synergic benefits to neurosensory retinal tissue which may manifest as visual performance enhancement with concomitant amelioration of digital asthenopic symptoms [199,200,201,206,246,247,249,268,280,282,283,295,325].

## 5. Conclusions

Computers, handheld electronic devices are now irreplaceable in our daily lives and has led to digital eye strain which is unfortunate unforeseen consequence. A comprehensive strategy for the management of digital eye strain must be tailored to reflect the complex etiology associated with the syndrome. The role of nutrition for promoting optimal visual performance and the potential implications associated with poor nutrient intake have become increasingly evident in recent years. In lieu of this, current literature offers promising evidence that adjunctive nutraceutical strategies may confer additional ocular and systemic health benefits for individuals experiencing digital eye strain.

## Figures and Tables

**Table 1:** Characteristics of the randomized clinical trials using omega-3 PUFA.

Author (Year)	Participants	Duration	Interventions per Day	Results
Bhargava (2015) [171]	478 patients with CVS; aged (23.3 ± 4.7) years in India	3 months	360 mg EPA + 240 mg DHA; placebo	Significant improvements in TBUT, Schirmer scores, and DESS scores (*p* < 0.01, for all)
Bhargava (2016) [178]	266 patients with CVS; aged (2±9.4 ± 4.8) years in India	45 days	1440 mg EPA + 960 mg DHA; placebo	Significant improvements in TBUT (*p* < 0.01) and DESS scores (*p* < 0.001)
Deinema (2017) [172]	54 patients with mild/moderate DE; aged (42.6 ± 3.9) years in Australia	90 days	1000 mg EPA + 500 mg DHA (in fish oil); 945 mg EPA + 510 mg DHA (in krill oil); placebo	Marked reduction in tear osmolarity (*p* < 0.001) and improvements in tear film stability (*p* < 0.05)
Epitropoulos (2016) [173]	105 patients with DE & MGD; aged (56.8 ± 17) years in USA	12 weeks	1680 mg EPA + 560 mg DHA; “placebo” (3136 mg linoleic acid)	Statistically significant reduction in tear osmolarity, OSDI scores, and TBUT (*p* < 0.01, for all)
Kangari (2013) [170]	64 patients with DE; aged (61.2 ± 8.3) years in USA	1 month	360 mg EPA + 240 mg DHA; placebo	Remarkable improvements in TBUT (*p* < 0.001), Schirmer’s scores, and OSDI (both *p* < 0.05)
Korb (2015) [174]	26 patients with Evaporative DE; aged (41.7 ± 19.8) years in USA	3 months	1000 mg omega-3 PUFA; placebo	Mean OSDI scores improved (+55%) significantly from baseline (*p* < 0.001)
Macsai (2008) [175]	38 patients with MGD; aged (50.7 ± 9.1) years in USA	12 months	~3300 mg ALA (in flaxseed oil, 6 g); placebo	Significant improvements in meibum scores (*p* = 0.003), TBUT (*p* = 0.002), and omega-6 to omega-3 PUFA ratio in plasma and RBC (both *p* < 0.05)
Malhotra (2015) [176]	60 patients with moderate MGD; aged (53.3 ± 6.9) years in India	12 weeks	720 mg EPA + 480 mg DHA; placebo	Enhanced benefits in OSDI scores, TBUT, and CS (*p* < 0.05, for all)
Olenik (2017) [177]	61 patients with MGD; aged (mean 56) years in Spain	3 months	1050 mg DHA + 127 mg EPA + 90 mg DPA (1.2 g total); placebo	TBUT, mean OSDI scores, lipid margin inflammation improved significantly (*p* < 0.05, for all)

Abbreviations: CVS, computer vision syndrome; EPA, eicosapentaenoic acid; DHA, docosahexaenoic acid; TBUT, tear break-up time; DESS, dry eye scoring system; DE, dry eye disease; MGD, meibomian gland dysfunction; OSDI, ocular surface disease index; ALA, alpha-linolenic acid; DPA, docosapentaenoic acid.

**Table 2:** Characteristics of the randomized clinical trials using anthocyanin nutraceuticals.

Author (Year)	Participants	Duration	Interventions per Day	VDT-Task	Results
Kizawa (2021) [199]	44 adults with DES; aged (36.6 ± 9.1) years in Japan	6 weeks	200 mg bilberry extract (*multivitamin*); placebo	Video game (60 min)	Reversed adverse effect on pupillary response with VDT-task (*p* < 0.05)
Kono (2014) [200]	48 adults with eye strain; aged (52.8 ± 0.9) years in Japan	4 weeks	20 mg bilberry extract & 26.5 mg black soybean hull extract (*multivitamin*); placebo	*n/a*	Improved near-point accommodation variation in both eyes (*p* < 0.05)
Kosehira (2020) [193]	109 adults with heavy VDT use; aged (35.8 ± 7.0) years in Japan	12 weeks	240 mg standard bilberry extract; placebo	Video game (40 min)	Relieved tonic accommodation in ciliary muscle triggered by VDT-task (*p* < 0.05)
Ozawa (2017) [194]	88 adults with heavy VDT use; aged (30.7 ± 0.9) years in Japan	8 weeks	480 mg bilberry extract; placebo	Video game (60 min)	Marked improvement in CFF (*p* = 0.023) and subjective DES symptoms (*p* < 0.05)
Park (2016) [195]	60 adults with CVS; aged (38.9 ± 10.6) years in Korea	4 weeks	1000 mg bilberry extract; placebo	Watch movie (60 min)	Significant improvement in subjective asthenopic symptoms induced by VDT-task (*p* < 0.05)
Riva (2017) [196]	22 adults with heavy VDT use; aged (45.5 ± 7.3) years in Italy	4 weeks	160 mg Mirtoselect^®^ standard bilberry extract (≥36% anthocyanins); placebo	Video game (45 min)	Statistically significant improvement in Schirmer’s test score (*p* = 0.02)
Rossi (2021) [202]	30 adults with heavy VDT use; aged (44.9 ± 9.1) years in Italy	1 month	300 mg elderberry & 100 mg black currant extracts (*multivitamin*); control	*n/a*	Remarkable improvements in CVSS questionnaire scores and contrast sensitivity at higher spatial frequencies (*p* < 0.01, for all)
Sekikawa (2021) [197]	32 healthy adults with DES; aged (37.1 ± 8.4) years in Japan	6 weeks	43.2 mg bilberry extract; placebo	Video game (60 min)	Protective effect against accommodative function decline with VDT-task (*p* < 0.05)
Yamashita (2019) [198]	74 adults with DES; aged (44.8 ± 7.4) years in Japan	4 weeks	60 mg MaquiBright^®^ SMBE (≥35% anthocyanins); placebo	Video game (45 min)	Significant improvements in Schirmer’s test (*p* = 0.005), along with VAS and DEQS scores (both *p* < 0.05)

Abbreviations: DES, digital eye strain; VDT, visual display terminal; CFF, critical flicker fusion; CVSS, computer vision symptom scale; DES, digital eye strain; SMBE, standard maqui berry extract; VAS, visual analogue scale; DEQS, dry eye-related quality of life score.
